# Role-Aware Information Spread in Online Social Networks

**DOI:** 10.3390/e23111542

**Published:** 2021-11-19

**Authors:** Alon Bartal, Kathleen M. Jagodnik

**Affiliations:** The School of Business Administration, Bar-Ilan University, Ramat Gan 5290002, Israel; kathleen.jagodnik@biu.ac.il

**Keywords:** global information spread, information diffusion, local information spread, non-viral information spread, online social networks, role-aware analysis, social roles, user behavior online, viral information spread

## Abstract

Understanding the complex process of information spread in online social networks (OSNs) enables the efficient maximization/minimization of the spread of useful/harmful information. Users assume various roles based on their behaviors while engaging with information in these OSNs. Recent reviews on information spread in OSNs have focused on algorithms and challenges for modeling the local node-to-node cascading paths of viral information. However, they neglected to analyze non-viral information with low reach size that can also spread globally beyond OSN edges (links) via non-neighbors through, for example, pushed information via content recommendation algorithms. Previous reviews have also not fully considered user roles in the spread of information. To address these gaps, we: (i) provide a comprehensive survey of the latest studies on role-aware information spread in OSNs, also addressing the different temporal spreading patterns of viral and non-viral information; (ii) survey modeling approaches that consider structural, non-structural, and hybrid features, and provide a taxonomy of these approaches; (iii) review software platforms for the analysis and visualization of role-aware information spread in OSNs; and (iv) describe how information spread models enable useful applications in OSNs such as detecting influential users. We conclude by highlighting future research directions for studying information spread in OSNs, accounting for dynamic user roles.

## 1. Introduction

**Figure 1:**
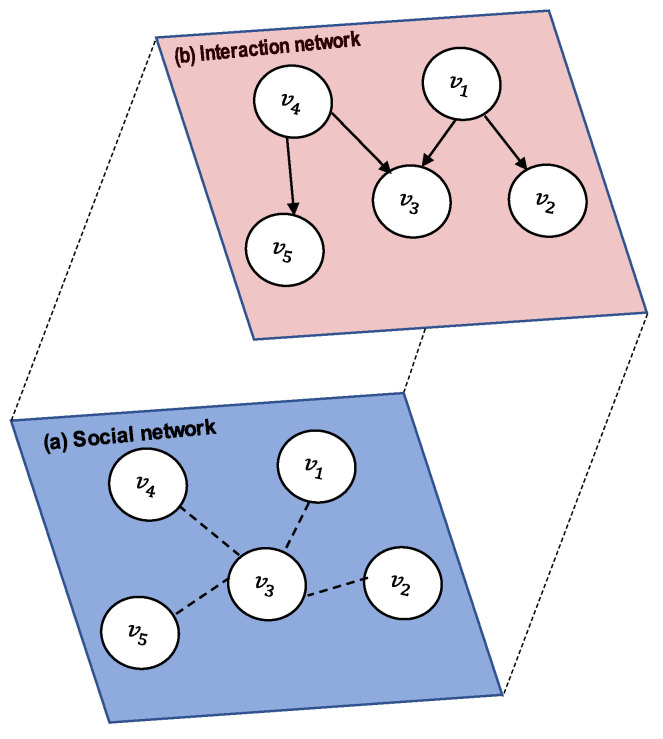
Illustration of two types of networks with the same set of nodes and different edges (links): (**a**) a social network in which dashed edges represent social relationships among users (e.g., Facebook friendships), and (**b**) a directed interaction network laid over the social network, in which solid edges represent user interactions (e.g., user $v_{4}$ retweeted a message originated by $v_{3}$ and another message originated by $v_{5}$).

Information spread is the process by which a piece of information reaches different individuals [1,2]. The ways in which people consume and share information have dramatically changed due to the rapid development of online social networks (OSNs) that enable their users to interact worldwide by, for example, publishing messages, sharing experiences, and exchanging opinions [3,4]. Some OSNs support two types of networks: (i) a *social network* is typically represented as a graph in which nodes represent users, and edges (links) represent social relationships (e.g., friendships on Facebook); and (ii) an *interaction network* describing how the information spreads, in which nodes represent users in the social network who adopted the information and edges represent interactions among users (e.g., posting a personal message, re-posting information, or liking a post). Laying the interaction network over the social network (Figure 1) creates a multi-layer network with multiple types of edges, defined as a tuple <V,E,L> where: *V* represents nodes; *E* is a set of tuples $<v_{i},\;v_{j},l>,\;v_{i},\;v_{j}\in V,\;l\in L,\;v_{i}\neq v_{j}$ and for any two tuples $<v_{i},\;v_{j},\;l>,\;<v_{i}^{\prime },\;v_{j}^{\prime },\;l^{\prime }>\in E$ if $<v_{i},\;v_{j},\;l>\neq <v_{i}^{\prime },\;v_{j}^{\prime },\;l^{\prime }>,\;v_{i}=v_{i}^{\prime }$ and $v_{j}=v_{j}^{\prime }$ then $l\neq l^{\prime }$; and *L* is a set of distinct layers (types of relationships).

The spread of information in OSNs mainly relies on interactions among users [5], with local user-to-user diffusion over the social network as a key mechanism found in the spreading of information in networks describing technological innovations [6], product purchasing in viral marketing [5,7,8,9,10], and adoption of opinions [1]. Thus, understanding information spread in OSNs is essential for a variety of applications, including controlling the spread of rumors [11] and monitoring public opinions [12].

A piece of information can spread within minutes to millions of people via online social interactions [13]. The spreading speed and reach size of the information in OSNs is affected by factors including information sharing of highly connected users [14] and their motivation in sharing the information based on their interests [15], which implies a variety of user social roles. An OSN user who decides to share a piece of information posted by another user (e.g., a retweet on Twitter) is considered influenced [16,17,18,19]. The influence of one user on another is a complex process that depends on many factors, such as user personal interests, reflected by the content of a message [16,20]; and users’ attributes, for example, resulting from network position and age [21,22,23,24,25,26,27]; these attributes are often referred to as user roles. To better understand information spread processes such as the spread of misinformation [28,29], researchers have tried to model information spreading paths resulting from influence [16,19,30,31,32].

The online behaviors of users determine their social roles and affect how information spreads in OSNs [33]. Highly active users have a potential to spread information quickly to multiple people [31] and influence them to spread the information, as well. For example, it was found that half of the URLs posted on Twitter were tweeted by a highly active minority of users (fewer than 1%) who act as opinion leaders [34]. Those influence events that result in the spread of information produce dynamic changes in users’ online behaviors that are reflected in the structure of the network [31,35,36] and can be modeled by learning the interaction characteristics among OSN users [31]. For example, a User *A* who was exposed to interesting information originated by another User *B*, might decide to follow User *B*, thus forming a new edge in the social network. In addition, User *A* might be influenced to share the information originated by User *B*, thus creating a link in the interaction network. To better explain the observed information spreading patterns in both types of networks, it is essential to identify the categories of social roles of individuals who affect the information spreading process.

Studies on modeling information spread in OSNs can be categorized into three main approaches of (i) structure-based models; (ii) non-structural models; and (iii) hybrid models. Structure-based models utilize the structure of the social network to infer information spread. These models include, for example, the Linear Threshold Model [37] in which network edges are weighted, and a user can transition between non-active and active roles if the sum of incoming edge weights from active neighbors exceeds a threshold. Using structure-based models, the probability that a user who was exposed to a piece of information will share it depends also on the social roles of the neighbors [33]. In contrast to earlier decades when the mainstream media (e.g., TV and radio stations) were the main sources of information, currently, people use OSNs to consume information [3,4] posted by other users, not necessarily neighbors in the social network, as well as information promoted by content recommendation algorithms and retail publishers [20,27,38]. For example, the Twitter Home timeline exposes a user to content posted by his/her followers, but also to promoted content and personalized recommended content originated by non-neighbors that Twitter thinks will interest the user. This mechanism of sharing global information, not limited to node-to-node spread, is addressed in non-structural based models [39,40,41] that infer the spread of information by non-structured mechanisms (e.g., homophily [42,43]) other than node-to-node propagation. This means that the role of a user in spreading information via OSNs is not restricted to detecting similar local connections. Thus, users can be affiliated with the same role even if they are remote and unconnected. Extending the idea of detecting users with the same role who do not share structural network similarity (e.g., using homophily), topic-aware information spread models have been utilized as non-structural models to identify users who interact with a similar topic distribution, under the assumption that similar interests imply similar behavioral patterns (i.e., roles) [15]. Hybrid models simultaneously consider structural and non-structural mechanisms to explain information spread. For example, Myers et al. [27] developed a model for explaining how information spreads in OSNs by considering both local node-to-node propagation and external information sources.

A systematic review of the literature about information spread in OSNs reveals the following limitations of existing models that call for future research. First, structure-based models utilize the structure of the network and incorrectly assume that information can diffuse only via the edges of a social network. In addition, these models ignore structural differences between users that imply user roles, neglect personal preferences or interests of users (e.g., Zhang [44]), and omit global information spread mechanisms by non-neighbors (e.g., exposure to information by mainstream media sources). Second, non-structural models consider spreading mechanisms beyond local user-to-user propagation but ignore the topology of the network and only consider the global rate of information spreading patterns [45]. Several studies addressed the challenge of modeling both approaches (structural and non-structural) in a single model by creating hybrid models that combine both local and global features. For example, [46] generates structural features by analyzing the social network or the interaction network, and non-structural features by analyzing topics resulting from user exposure to broadcast news, posts from friends, and users’ interests [15]. However (third), hybrid models to date have failed to integrate rich features that affect the complex process of information spread such as local spread mechanisms, global spread mechanisms, the context of a message, social roles reflecting personal user behavior, and personal user interests, leaving much room for improvement. Although several techniques [33,46,47] have been developed for modeling information spread by extracting latent topics of posts and hidden user social roles, accurately characterizing the dynamic role-aware, topic-dependent process of information spread is still not fully understood. Lastly, the fourth limitation of most recent information spread studies in networks (e.g., [22,23,24,25,48,49,50,51,52]) is related to focusing on the spread of viral information that leads to influence, but largely ignoring the spread of non-viral information. For example, Nahon et al. [53] studied the viral spread of the "Yes We Can" slogan in the 2008 U.S. presidential election in social and mainstream media first by opinion leaders and then by other users. In addition, there is a lack of consensus among viral information spread studies about the number of influence events that define an information as viral. The number of users who are influenced by a piece of information follows a long-tailed distribution [22,54]. Thus, most information nuggets are shared only a scant number of times [17,20,22,55], and many information nuggets are not shared at all. This finding implies that influence resulting from viral versus non-viral information spread follows different infection mechanisms [19] and necessitates future research about the spread of non-viral information.

Some of the presented gaps have been addressed in recent review papers on information spread in OSNs [12,32,56,57], covering algorithms for modeling the local node-to-node spread of viral information. However, these reviews present three main gaps. First, previous reviews have not fully considered the roles of users in the spread of information. Second, neglecting to address global information spread beyond OSN edges (links) via non-neighbors through, for example, information pushed via content recommendation algorithms; Third, neglecting to address the spread of non-viral information with low reach size. To address these gaps from previous review papers on information spread in OSNs, this review paper contributes by: (i) presenting a comprehensive survey of the latest studies on role-aware information spread in OSNs, including the different temporal spreading patterns of viral and non-viral information; (ii) surveying modeling approaches that consider structural, non-structural, and hybrid features, and providing a taxonomy of these approaches; (iii) surveying software platforms for the analysis and visualization of role-aware information spread in OSNs; and (iv) describing how information spread models facilitate useful applications in OSNs such as detecting influential users. We conclude by presenting future research directions for studying information spread in OSNs, accounting for dynamic user roles.

Organization. In Section 2, we cover literature about social roles, followed by a survey of computational models aimed at discovering social roles in Section 3. Section 4 addresses the *first* gap in existing review papers of not fully considering user roles in the spread of information, by discussing how user social roles affect information spread. We also address the *second* gap in existing reviews involving their coverage of only node-to-node information spread mechanisms, by reviewing structural, non-structural, and hybrid information spread models in OSNs. More specifically, we provide a brief overview on information spread including its mechanisms, the motivation for modeling information spread in OSNs, and how exposure to information can lead to influence that affects users’ behavior and network structure. In Section 5, we address the *third* gap in existing review papers of their covering only viral information spread by describing and comparing viral vs. non-viral information spread models. Section 6 reviews software platforms for exploring user roles in OSN information spread. Finally, we conclude this review and share our perspectives regarding future research in Section 7.

## 2. Social Roles

Social roles were first identified by sociology research [58] and have been used to categorize the behaviors of individuals. Early definitions of the term *role* include, for example, “a cluster of related and goal-directed behaviors characteristic of a person within a specific situation” [59], and “behavioral expectations of a person” [60]; for example, a person holding the role of a teacher is expected to be an educational figure. Moreover, it is expected that different individuals with the same role behave in the same way. In the physical world, outside OSNs, role definitions sometimes include a formal job description with a list of responsibilities [61]. Biddle [62] defined five major types of social roles, each described by a developed theory: (i) Functional Role Theory—focuses on social roles with an emphasis on the characteristic behaviors of the people who serve in these roles; (ii) Symbolic Interactionist Role Theory—assesses how individuals interpret observed behavior differently and how they affect each other’s behavior via interactions; (iii) Structural Role Theory—utilizes mathematical models to examine the influence of the society as a whole on role selection by individuals; (iv) Organizational Role Theory—examines role development in organizations; and (v) Cognitive Role Theory—analyzes an individual’s expectations of a person holding a role compared with the observed behaviors of that person.

In recent years, especially during the restrictive regulations of the COVID-19 pandemic, people have increasingly used OSNs to maintain social relationships and interact globally [63]. Some of these OSNs allow the formation of virtual groups of people (online communities) who interact with each other and share common values, beliefs, behaviors, interests, personal preferences, and other characteristics [64,65]. In most online communities, the majority of users never contribute (90% in [66]), a small group of users account for almost all network activity (1% in [66]), and the rest of the users (9% in [66]) contribute minimally, which implies the existence of user roles. For example, *influentials* are a small minority group of very active users affecting information spread in OSNs [67] who influence multiple users to be active, as well [68].

Studying user roles in OSNs can improve our understanding of how individuals contribute to their community and spread information [59]. The importance of roles is acknowledged in many applications such as predicting new network links [69] and modeling dynamic user behavior in large graphs [70,71]. The growing usage of OSNs establishes the need to better understand user behavior online to recognize emergent behaviors characterized as social roles that define user contributions in generating and spreading information.

Several studies have identified the distinct roles of OSN users via their activity levels in OSNs [31,72,73]. A *visitor* is a user who consumes information, but who does not participate in discussions or interact with other users [31]. A user who participates in discussions is designated as a *novice* [31]. With frequent participation, as well as the activities of viewing and generating content in the community, the novice is upgraded to the *activist* role [31]. An activist earns the designation of a *leader* in a community, also referred to as a *gatekeeper* [73], by engaging in the role of an opinion maker who influences many other community members to engage in similar activities. In the opposite direction, a user becomes *passive* if his/her activity is limited to maintaining interest in discussions and in other users, only consuming content rather than generating it. Finally, a *troll* causes conflicts with other users by posting offensive content, typically engaging in such antisocial behavior over a relatively short period, prior to disappearing from the community [31,72].

While OSN users can be assigned formal roles, such as administrators [74], more often, roles in OSNs are not formally defined, thus challenging other OSN users to identify and understand the roles and behaviors of their community members. Many studies [68,70,75,76,77] have reported roles that emerge from the structure of the network and that can be identified via network centrality metrics (e.g., Indegree, Outdegree, Closeness, Betweenness, Eigenvector [75]) or ranks (e.g., PageRank, HITS), which reveal structural information about user relationships within the community [78]. For example, when analyzing the 9/11 terrorist attacks [79], influencers who served in a variety of roles (e.g., “gatekeepers” and “brokers”) were identified using centrality measures. Additionally, centrality measures have been employed to assess the importance of users in networks [80] (e.g., via articulation nodes and selection of the top-k nodes [81]). In contrast, other studies [82] have identified no correlations between user roles and their structural positions. Such findings might result from the analysis of dynamic user behavior online as in [31], which identifies more nuanced roles than simple analyses of a single, static snapshot of the network, when considering whether a user is highly influential or not. Additionally, since OSN users often change roles [31,83], it is important to use the dynamic structural relations of users in order to identify diverse, shifting roles, as reviewed in Section 3.

Each role in an OSN has a unique signature that can be defined by observing the patterns of user participation [84]. These patterns typically include structural network centralities such as Closeness [85], Degree [86], and Betweenness [87], or changes in node embeddings (a method of representing nodes as vectors) [88] over time, but can also include temporal patterns such as the time interval between posts [20]. Users’ interactions change and evolve over time [31,89], and can be characterized as roles that describe user behavior [31,90]. For example, a user who interacts with users who affiliate with two isolated communities is considered a mediator. The underlying assumption of role detection models is that users who affiliate with the same role have similar network structural patterns [70]. This assumption does not require that users with the same role will be neighbors or close in the network [69,77]. Moreover, a user can play different roles and affiliate with different communities [69]. Users who affiliate with the same community must be close in the network, whereas users who affiliate with the same role can be far from each other. Recent studies utilize the structure of a network to define a community as a group of users who are highly connected among themselves and have fewer connections outside the group [58]. By analyzing the structure of the network, we can identify different communities and their affiliated members [69,91] and roles [58,92].

The association of a single affiliation of a member to a community for a specific snapshot of the network was expanded to allow community detection algorithms to simultaneously assign individual users to several communities [93]. For example, the Bayesian Hierarchical Latent-Factor model (BHLFM) was designed to uncover hidden affiliations of members to multiple communities and roles [69]. Users who simultaneously affiliate with several communities in the analyzed network are considered to have an important role due to their structural location in the network that enables them to potentially control the spread of information among those communities [94]. These users who affiliate with several communities have served central roles in increasing the spread of information reach about presidential campaigns as well as propaganda [95].

Identifying important users in networks is essential for understanding and managing the flow of information in OSNs. This strategy has been employed to contain or extinguish outbreaks of epidemic infections [96]. For example, during the COVID-19 crisis, governments have needed to identify superspreaders of the SARS-CoV-2 virus and isolate them to contain the disease spread [96]. Similarly, identifying important users in OSNs is necessary for delaying or preventing the spread of misinformation in OSNs [97,98]. For example, a small minority of 0.1% of Twitter users (superspreaders) were found to be responsible for spreading 80% of fake news [99]. Aiming to locate users with a significant potential to control the spread of information, Chang et al. [100] developed a model to identify a small minority of nodes describing unique physical locations that act as superspreaders, accounting for the majority of infections. Since these superspreader and influence nodes have a massive influence on a large population [31], numerous studies have tried to identify them. Classic examples include detecting influential researchers in a citation network [101]; understanding user communication patterns in email communication networks [102]; identifying important junctions in road networks that might create bottlenecks [103]; and analyzing critical stop locations of flights between airports [104]. These studies analyzed nodes’ behavior to infer their informal (latent) social role, defined by the set of activities that users perform [105,106].

Sociological studies reviewed in the current section aim to describe and define social roles. As modern life becomes increasingly integrated with online systems, the concept of social roles becomes valuable as a tool for categorizing patterns of action, recognizing distinct user types, and cultivating and managing online communities. Utilizing sociological definitions of roles, computational models for role discovery in OSNs combine psychological, behavioral, and structural attributes for modeling user behavior online. Moreover, computational role discovery models often focus on the detection of latent roles using unsupervised methods, as elaborated next.

## 3. Role Discovery Models

Networks have been widely studied to learn complex interactions among users and discover their roles by analyzing the network structures [107]. Early network-based methods for role discovery used network centrality measures to identify user importance by, for example, selecting the top-k nodes with the highest measure [108]. For example, [109] proposed a weighted Degree decomposition method to rank and identify cheaters who can lead to the collapse of cooperation process in a dynamic evolving cooperation network. To improve node ranking, [110,111] computed the entropy of nodes using node Degree and the total Degree of a network. However, Degree centrality is limited to 1-hop neighbors only. Other network centralities such as Betweenness and Closeness centralities, when used for role discovery in networks, can become computationally extensive for medium and large-scale networks. In contrast, the k-core centrality [112,113] is much more efficient, in terms of consuming computational power, for role discovery in networks. k-core is an iterative graph pruning algorithm that groups nodes into layers, where the innermost core nodes have the highest k-core value and the outer nodes have the smallest k-core value. Hybrid k-core methods for node ranking for role detection use the k-core algorithm with a combination of other network centrality metrics such as Degree, Closeness, and Shortest Distance [85,86,87].

Utilizing network structure, recent studies use clustering [114] for detecting user roles in networks by affiliating users (nodes) to distinct or overlapping groups. The task of roles detection is complementary to the notion of community detection, which involves partitioning the network into cohesive subgroups. Network clustering has been extensively used for community detection [64,115,116,117] and role discovery [2,31,77] tasks. Community detection algorithms aim to find groups of users (nodes) that frequently interact with each other and have sparse interactions with other users not affiliated with the group [118]. Structural-based role discovery algorithms, on the other hand, group nodes based on their structural similarity [119]. For example, bridge and hub nodes [120] share structural similarity by connecting previously unconnected groups or users. The difference between the tasks of community and role detection is that users who affiliate with the same community are likely to be connected to each other, whereas users who affiliate with the same role can be distant in a network, and unconnected. Recent methods aimed to detect user roles in OSNs typically use unsupervised techniques to cluster structurally similar users together. The latent position cluster model [121] estimates unknown role affiliations by a set of probabilities of edge formation between users, where each user is associated with exactly one role.

Many role detection methods assume that user roles can be inferred by analyzing social interactions, and that users with similar network structural patterns affiliate with the same role [64,68,77]. Examples of models that utilize the structure of the graph to discover user roles include semi-supervised role inference models [122] and Blockmodels [123]. In another study [67], users’ social roles are identified by using the structure of the social network; it analyzed how the interactions between users with different roles impact information diffusion paths. The authors found five user roles in two categories of users’ ability to influence others in spreading the information, and in blocking (delaying) information spread. The single role affiliation assumption of the latter models was extended in the Mixed-Membership Stochastic Blockmodels (MMSB) [93] by allowing a user to affiliate with several roles. MMSB assumes a fixed network structure; however, OSNs are dynamic. Thus, dynamic MMSB (dMMSB) [124] and a few other models [31,69,91] consider network evolution, where users serve in various roles that evolve over time. The definition of roles in dMMSB implies that users with similar roles share common features and relation patterns, even without direct relationships [92]. More recently, network embedding methods [29,121,125,126,127] have gained popularity in studying graph structure, achieving state-of-the-art performance in downstream tasks such as node classification. These methods represent network data as vectors in a latent space to preserve the topological structure and properties of the original network. The most widely used network embedding methods can be categorized into (i) matrix factorization; (ii) random walk based methods; and (iii) deep learning models.

**Matrix factorization methods** [128], such as singular value decomposition (SVD) and non-negative matrix factorization (NMF), aim to learn node embedding via low-rank approximation and analysis of the adjacency matrix (denoted as *A*): a binary matrix describing a network, the elements of which $a_{ij}$ are 1 if there is an edge between these nodes, else 0, as defined in Equation (1).

(1) $$a_{ij}=\left\{\begin{array}{cc} 1, & \mathrm{if}\;\mathrm{there}\;\mathrm{is}\;\mathrm{an}\;\mathrm{edge}\;\mathrm{connecting}\;\mathrm{nodes}\;i\;\mathrm{and}\;j\\ 0, & \mathrm{otherwise}. \end{array}\right.$$

The REACT [129] algorithm generates embeddings via matrix factorization to identify both communities and roles. RolX [130] also utilizes matrix factorization. First, it computes primary node features (Degree, weighted Degree, and average Clustering Coefficients) using ReFeX [131] and recursively aggregates the features of a node’s neighbors. Second, NMF is used to generate node embeddings. GLRD [120] extends RolX by modeling role detection as a constrained NMF problem, where the guidance is provided as convex constraints and specified per role. RIDϵRs [92] first partitions the graph using the connectivity patterns among roles by applying the concept of ϵ-Equitable partitions, and then performs role discovery using NMF. GraphWave [132] is an unsupervised method for learning node embeddings based on structural similarity in networks. It treats graph diffusion kernels as probability distributions over networks and calculates embeddings by using characteristic functions of the distributions. HONE [133] analyzes motifs of a weighted graph where an edge’s weight is the count of the co-occurrences of the two endpoints in a specific motif. The main limitation of these matrix factorization methods is low computational efficiency resulting from calculating pair-wise node similarity.

**Methods based on random walks** capture node proximity to generate embeddings for capturing structural network similarity of nodes that are more likely to appear in the same sequence, and these methods map nodes with similar structural features to the same role. Regarding role detection, network nodes that affiliate with a role have the same embeddings. For example, struc2vec generates node features by performing random walks on a graph in which edges are weighted based on structural distances to capture structural information, assuming that nodes with similar network structure have similar roles [134]. Role2Vec [76] maps nodes into several disjoint roles using clustering methods that analyze node features and attributes such as K-means. Then, random walks are performed, and the generated sequence IDs are used as role indicators. struc2gauss [135] models both structural similarity by using RoleSim [136], and uncertainty using a Gaussian distribution. Each node is mapped to a Gaussian distribution, and the variance captures the uncertainties. SPINE [137] incorporates structural features of both local and global node proximity to learn embeddings. RiWalk [127] assumes that nodes with different functionalities have different roles in a network, and the structure of the network can be learned using random-walk-based node embedding. These methods reconstruct the edges between nodes based on the structural similarities so that the context nodes obtained by random walks are structurally similar to the central nodes. However, they do not jointly consider structure, content, and label information. struc2vec better represents role information than SPINE [138], which leads to better embeddings but results in higher complexities of computational time and space.

**Deep learning methods** for graphs were developed in recent years in an effort to optimize node embeddings representations. Therefore, to date, only a few studies [125,139,140] have leveraged deep learning methods for role-oriented network representation learning. For example, DRNE [125] develops a deep learning method with a normalized long short-term memory (LSTM) layer to learn regular equivalence by recursively aggregating neighbors’ representations for each node. GAS [141] utilizes graph neural networks (GNN) to capture node structure by applying sum-pooling propagation instead of the graph convolutional networks (GCN) [142] to better capture local node structures. For training, GAS extracts features similarly to ReFeX and aggregates them only once. Similarly, RESD [140] also relies on ReFeX [131] for generating features that are input to an autoencoder to learn node embeddings while reducing data noise during the learning stage. GraLSP [139] uses a GNN to learn node low-ranked representations by considering the node’s local structural patterns as well as the its neighboring nodes via random walks.

Deep learning methods have achieved state-of-the-art results in several graph-based downstream tasks such as node classification [143,144,145] and link prediction [145,146] that were not used for user role identification. Most deep learning methods mainly use autoencoders and GCNs that generally consist of an encoder, a similarity function, and a decoder. An autoencoder is a neural network with an encoder and a decoder architecture. The encoder takes a data point as input and converts it to a lower-dimensional representation. Next, the decoder takes this lower-dimensional representation and aims to reconstruct the original input as accurately as possible. A GCN is a convolutional network operating directly on graphs. For example, NetSMF [126] uses an autoencoder and graph convolutional networks to learn node representation vectors.

Among the three network embedding methods of matrix factorization, random walks, and deep learning, random walks perform best for small networks, whereas matrix factorization methods perform best for large networks with up to tens of thousands of nodes [147]. These methods analyze feature matrices that are characterized by having lower dimension than the original graph matrices analyzed in the random walk-based methods. Deep learning methods scale well for huge networks having millions of nodes, outperforming other methods for role discovery [138]. Huge networks contain multiple examples that permit sufficient training of deep learning models, thus allowing them to achieve excellent performance. In addition, neural network graph-based models work well when nodes are labeled by their structural roles (structure aware). However, they fail when nodes are labeled by their position in the graph (e.g., community affiliation) since they have the same embeddings.

A few studies have identified roles in large dynamic networks. For example, the Role Affiliation Frequency Model (RAFM) [31] considers dynamic networks in which each user is affiliated with a role at each time step. Thus, a user can play multiple roles. RAFM assumes that users’ latent behavior has a different blend of social roles (e.g., a user can be a novice but also have the qualities of a leader) that can change over time. Gupte and Ravindran [71] used MapReduce to identify roles in large sparse graphs with only three time steps. Rossi et al. [148] generated a role transition model per user where role meaning is defined by network centralities. Other studies for role detection in OSNs analyzed text inserts of millions of users, where user role is determined using different word selections, as discussed next.

**Textual role discovery models** based on Natural Language Processing (NLP) can be used to automatically identify social roles in OSNs [149] via analysis of the latest user textual interactions that capture the most up-to-date behavioral cues in users’ language [150]. For example, [151] learned roles in movies and novels from the language of plot summaries and dialogue. Welser et al. [152] identified four roles in Wikipedia users: substantive experts, technical editors, vandalism fighters, and social networkers, by analyzing users’ textual inserts. Fazeen et al. [153] classified Twitter users into laders, lurkers, associates, and spammers by analyzing user tweets. Yang et al. [106] analyzed the editing behaviors of Wikipedia users and applied a Latent Dirichlet Allocation (LDA)-based model to detect editor roles. Ferschke et al. [154] found four roles: workers, critiquers, encouragers, and managers, by applying a similar approach to [106]. Maki et al. [155] proposed a supervised graphical model with an outcome measure to define editor roles based on interactions of users who contributed content on Wikipedia. Other role studies that analyze texts identified experts of a topic [156,157], opinion leaders [158], and influential bloggers who are not necessarily the most active users, by measuring the lengths of posts [75]. Recently, analysis of health discussions online revealed that the presence of a moderator user led to increased user engagement [159]. Users who continuously contribute rich content can strongly affect information spread in OSNs and influence other users to contribute.

**Figure 2:**
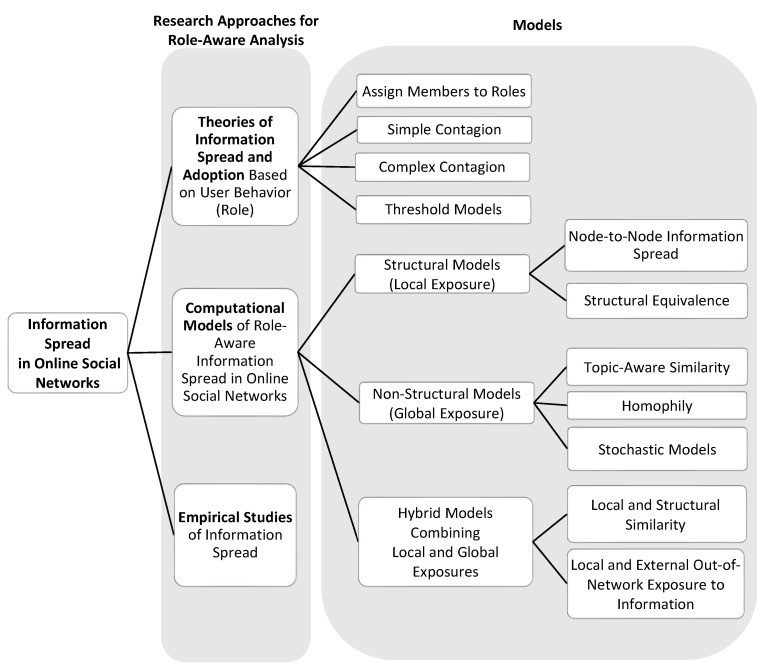
High-level taxonomy of analysis strategies for information spread in online social networks. Grey panels group research approaches for role-aware analysis (left panel), and the associated models (right panel). Refer to Section 4 for a list of studies exemplifying structural models, non-structural models, hybrid models, and models employing external information.

To summarize, most role detection studies focus on (i) estimating users’ importance by ranking them based on their capability to spread information; and (ii) finding a minimal set of users to maximize information spread in terms of reach size, often referred to as the Influence Maximization problem [160,161,162,163]. The spread of information in OSNs that leads to influence is discussed in detail in the following section with an emphasis on user roles, where we review approaches and models of information spread in OSNs, discuss their shortcomings, indicate open questions, and summarize them in a taxonomy (Figure 2).

## 4. Role-Aware Information Spread in Online Social Networks (OSNs)

**Figure 3:**
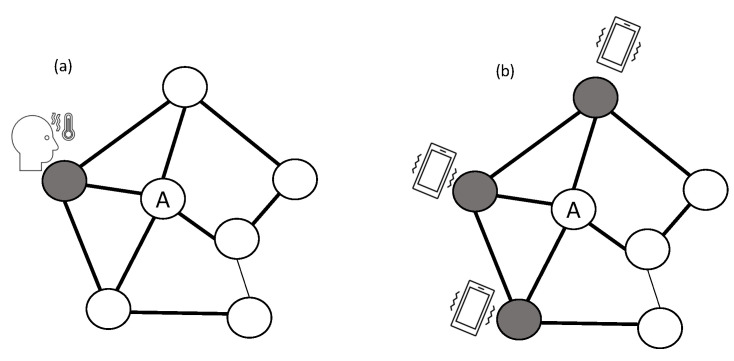
An illustration of simple and complex contagion in a social network. (**a**) Simple contagion: node *A* was infected by a disease after exposure to a single infected node (colored in gray). (**b**) Complex contagion: node *A* adopted a product (a smartphone) after being exposed by three nodes (colored in gray) who adopted the product.

The widespread usage of OSN platforms such as Facebook, LinkedIn, Sina Weibo, Tumblr, and Twitter enables information to spread quickly. Exposure to information might influence the exposed user to engage by, for example, re-sharing the information [16,19,20]. Relying on the structure of the network, some studies on the spread of information assume that ideas and behaviors have similar spreading mechanisms as diseases [35]. However, these models cannot fully explain the observed spreading patterns of information in OSNs that leads to influence (also referred to as contagion, or adoption) [27,164,165], such as complex contagion. Infectious diseases typically follow *simple contagion*—a contagion probability that is independent of the number of exposures between a source who was infected and a non-infected entity [166]. However, in OSNs *complex contagion* is also at play—a contagion probability that requires multiple exposures to one or more infected entities [166]. Complex contagion is more successful at describing the spread of ideas, technologies, or behaviors than simple contagion [166,167]. Figure 3 illustrates simple and complex contagion.

OSN users who have a strong influence on others typically hold the role of opinion leaders who can quickly and efficiently spread information. Many researchers have pursued the identification of opinion leaders in social networks [68,70,75,76,77,95,168,169] by calculating centrality measures (Table 1). Identifying opinion leaders is important for understanding how information spreads in events including political campaigns [170,171], the adoption of new technologies [172], and planning online marketing and advertising [173]. A research area related to the detection of opinion leaders is the Influence Maximization problem [160,161,162,163], introduced in Section 3, that involves selecting a minimal set of unique nodes that will maximize the spread of information and has an NP-complete complexity [163].

**Figure 4:**
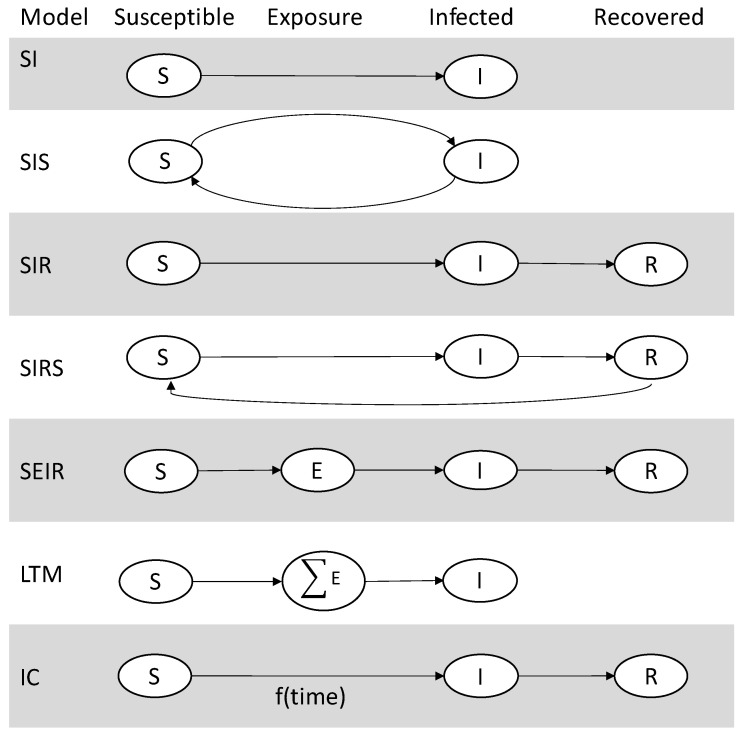
An illustration of the models Susceptible-Infected (SI) [187], Susceptible-Infected-Susceptible (SIS) [187], Susceptible-Infected-Recovered (SIR) [188], Susceptible-Infected-Recovered-for-Susceptible (SIRS) where immunity lasts for only a short period of time [187], Susceptible-Exposed-Infected-Recovered (SEIR) [189], the Linear Threshold model (LTM) for influence maximization [37], and the Independent Cascade model (IC) [190]. In the LTM, a node is exposed to its neighbors, and if the number of infected neighbors exceeds a threshold, the exposed node is infected. In the IC model, each infected node stays active during one time step only and tries to infect its susceptible neighbors with a certain probability. The attempts are independent random events. A susceptible node that was infected will attempt to infect its neighbors at the next time step.

Numerous studies [68,70,75,76,77] have aimed to identify central users who can best spread information quickly and efficiently. Other studies have focused on modeling the spreading process of information instead of finding top spreaders. These models typically aim to predict the next node in a network that will share a piece of information (i.e., become infected) such as the Susceptible-Infected (SI) [187], Susceptible-Infected-Susceptible (SIS) [187], Susceptible–Infected–Recovered (SIR) [188], Susceptible–Infected–Recovered–Susceptible (SIRS) [187], Susceptible–Exposed–Infected–Recovered (SEIR) [189], the Independent Cascade model (IC) [190], and the Linear Threshold model (LTM) for influence maximization [37] that are summarized in Figure 4. The mechanisms behind those models will be covered in the following subsections.

Users’ tendency to stay active by creating and sharing information (e.g., a retweet on Twitter or a share on Facebook) can be employed to predict user roles [31] that affect the spread of information [68]. OSN users’ motivation to act is affected by, for example, their will to interact with other users or to express interest in the discussed content [191]. Online interactions affect the reach size (often termed *cascade*) and the speed by which information spreads [192]. For example, online interactions by followers of celebrities on Twitter led to multiple retweets of content published by those celebrities [193]. As illustrated in the taxonomy provided in Figure 2, information spread models can be classified into three main groups of (i) Structural, (ii) Non-structural, and (iii) Hybrid information spread models that combine characteristics of both of those model classes, as elaborated next.

### 4.1. Structural Information Spread Models

Users have different motivations for spreading information [27,194,195], and these motivations inform the study of social behavior as expressed as OSN user roles. The study of information spread in OSNs typically assumes that a User *A* who follows another User *B* is more likely to spread information created or shared by User *B* than users who do not follow User *B*. In other words, structural-based models try to model the spread of information by assuming the existence of underlying node-to-node spreading mechanisms [19,20]. Based on the assumption that information spreads from node to node over network edges, recent studies using structure-based models [19,196] analyzed the social network of a user to infer the spread of information. Several models have been proposed and studied, demonstrating that the structure of the network affects user behavior and activities [197,198,199,200]. For example, the cohesion model [199] is an empirical model measuring the time of adoption to explain the phenomenon of physicians innovating to prescribe the drug tetracycline to patients after directly communicating (cohesion) with other physicians. The authors of [199] detected the time the information was shared by considering the time a prescription was written and found that highly connected physicians in the social network wrote a prescription more rapidly than those who have sparse connections with other physicians, implying different user roles. Valente [197] also presented an empirical model with which he analyzed the time of adopting an innovation.

Similarly to real-world social networks, in OSNs, network structure can be utilized to detect user-to-user exposure to information that can lead to the spread of this information. For example, Kramer et al. [201] found that the emotions of Facebook users that were seen by their friends via the Facebook Wall feature led the exposed friends to express similar emotions, thus influencing those users to spread emotional information online. Bakshy et al. [21] found that exposure of a user to information posted in a URL on the Facebook feed (that presents his/her Facebook friends’ activities) increases the user’s likelihood to spread that information by sharing the URL address. Sun et al. [202] studied how exposure to information about online activities of a neighbor can influence the online participation of users in fan pages.

The spreading patterns of information via a user-to-user mechanism were found to generate a tree-like structure [22,164]. For example, Kleinberg [190] suggested a structural transition model with user-to-user information spreading mechanisms that creates a tree-like structure, and analyzed the local spread of information by allowing a user to activate inactive nodes at a distance of 1-hop (neighbors) in the network. Two-step diffusion models [164,203] use a slightly different modeling approach in which information first spreads from the mainstream media to opinion leaders. Then, it spreads using a local user-to-user mechanism from opinion leaders to a broader population and creates a tree-like structure.

In retail marketing, exposing people to information via traditional advertising, such as radio, TV, and newspaper commercials, is becoming less effective in convincing people to buy a product [4]. Thus, retailers have been engaging in numerous efforts to convince customers who bought a product to expose their neighbors in OSNs to their experience by recommending other users to buy that product [8]. For example, Leskovec et al. [204] studied a product recommendation network, measuring the extent to which exposure to information about a user’s activity in recommending a product can influence their network neighbors to purchase that product. Many other examples of OSN users who exposed their neighbors to information about product usage exist, such as the Old Spice “The Man Your Man Could Smell Like” campaign [9], the ALS Ice Bucket Challenge [26], and Coca-Cola’s “Friendly Twist” campaign [10]. According to Rogers’ Diffusion of Innovations theory [205], people can be assigned to roles (see taxonomy in Figure 2) of Innovators (2.5%), Early Adopters (12.5%), Early Majority (34%), Late Majority (34%), and Laggards (17%) based on how quickly they adopt an innovation.

Following the notion of categorizing users into roles, threshold diffusion models in OSNs define groups (roles) of users so that users who are affiliated with different groups have unique behavior with varying thresholds of adoption [206]. In other words, users are influenced to adopt a behavior or an innovation depending on the number of their OSN neighbors who were influenced. For example, Centola [207] analyzed the number of exposures to information about health behaviors of neighbors in an OSN that are required for a user to adopt a health behavior. The LTM [208] also utilizes user-to-user information spread with a threshold criterion by focusing on a user’s tendency to be influenced resulting from exposure to information by neighbors. The LTM was expanded [209] to consider the temporal order of influence events by considering the time window in which a user can be influenced, revealing that user bursty activities are able to explain information spread in OSNs.

The structural information spread models reviewed so far in the current section are limited by their inability to consider the spread of information beyond 1-hop neighbors such as user exposure to promoted content. A few studies have addressed the limitation of local user-to-user information spread modeling. For example, the structural-equivalence model [210] considers exposure to information by analyzing the extent to which two users are connected to the same other users in a network. The structural-equivalence model argues that the larger the overlap between user *A*’s set of neighbors and user *B*’s set of neighbors, the more likely user *A* and user *B* will be exposed to similar information [210]. Following the structural-equivalence model, network users should be able to discern the overlap of their neighbor sets and also be able to access each other’s activities. However, it is unlikely that users who are not neighbors in a network can observe the overlap of their neighbor sets based on network structure alone. Addressing this problem, Leenders [211] states that the overlap between the neighbors of two network users can be assessed at a sociometric distance of maximum 3-hops.

Structural models account for information by user-to-user exposure mechanisms, which consider only local information spread sources at a distance of 1-hop (e.g., the cohesion model [199]), and the structural-equivalence model considers a wider exposure to information sources at a distance of at most 3-hops. Taken together, those structural information spread models cannot infer the spread of information from users who are located at more remote distances. While in the past, human interactions were limited by physical proximity, currently, with the wide use of the Internet and the increased usage of OSNs, the lack of physical proximity is no longer a constraint for interacting [206].

Recently, numerous studies have modeled information spread by analyzing the diffusion paths of information and using the structure of a social network to predict the next node that will spread the information [212]. Many of these analyses have used neural networks [88,213,214]. For example, [215] applied a recurrent neural network (RNN) to jointly predict the next infected user and estimate the reach size of infected users.

Models that aim to explain information spreading paths that account for user roles by learning user representation typically analyze information spreading patterns as sequences of data. For example, the TopoLSTM model [88] built dynamic directed acyclic graphs (DAGs) consisting of information spread paths and extended Long Short Term Memory (LSTM) network to learn the DAG structure in order to automatically learn user representation. CYAN-RNN [214] applied an attention mechanism to the spreading patterns of information represented using a tree-like structure to capture cross-dependencies of information spreading sequences. Similarly, the DeepDiffuse model [216] used an attention mechanism together with embeddings to predict the time of the next infection and the user who will be infected next.

Most of these models are limited by ignoring the impact of the changing preferences of users over time. To address this limitation, Cao et al. [217] developed a dynamic structural-temporal graph neural network (DySTGNN) that considers both the structure of the social network and temporal features in the information spread graph. Applying a graph attention network (GAT) to the information spread graph, the authors learn user embeddings representing short-term preferences of users. Then, they employ a graph convolutional network (GCN) to the social network and learn structural embedding for each user.

With the phenomenon of global exposure beyond local exposure to information, the mechanisms for online information spread have substantially changed during the last two decades and, thus, information spread has been considered beyond the structure of a network. The local user-to-user exposure to information that is the basis of structural information spread models misses the global mechanisms by which OSN users are exposed to varied content by non-neighbors, beyond network structure, that can lead to global information spread [27,68]. On Twitter, for example, users can be exposed to hashtags that are posted by non-neighbors [218] as well as to promoted information on a user’s Home timeline [219] that contains tweets of followees as well as information tweeted by non-neighbors either by purchased advertisements or tweets ranked as having a large engagement potential [220]. Reddit and Facebook are other examples of network services that allow global exposure via trending topics that appear on a user’s front page [221]. Therefore, beyond the local exposure to information by network neighbors, it is important to consider global exposure to information by non-neighbors when modeling information spread in OSNs, as discussed in the next subsection.

### 4.2. Non-Structural Information Spread Models

In contrast to structural information spread models, non-structural models largely ignore the structure of the network to infer the spread of information that can lead to influence, adoption, or contagion [41,222,223]. Two commonly studied contagion spread models in epidemiology are named SIR and SIS [224] (Figure 4), where nodes with the *S* role are Susceptible, the *I* role are Infectious, and the *R* role are Recovered. In the SIS model, individuals can transition from the role of *S* to *I* and back, and in the SIR model, individuals can transition across the roles *S* to *I* to *R*. SIR and SIS assume that every user has the same random probability to become infected, i.e., users have the same contact rate that is indicated by an edge formation in a network.

Although the SIR and SIS models fail to accurately explain biological contagion processes, particularly at large scales [225], those paradigms have been used to model the adoption of ideas [226], diffusion of innovations [227], and the spread of rumors [11]. Extending the SIS model, Leskovec et al. [204] propose an SIS model in which all users share the same probability β to adopt a piece of information. Users who adopted the information hold the susceptible role at the following time step. Since the assumption that influence is evenly distributed among OSN users is not valid [32], more complex modeling strategies such as exposure rates [16,27] have been developed. For example, Myers et al. [27] measured the level of exposure to information using hazard functions that facilitate exposure curves, such that each new exposure increases the probability of influence.

Other OSN studies that used non-structural models to explain information spread that leads to influence include, for example, the Linear Influence Model (LIM) [39], in which the influence functions of users are affected by the overall rate of influenced users in the network. LIM assumes a static network structure, and integrates exposure effects from a single source at each time step. The Heat Diffusion model [228] assumes a similar logic between heat diffusion in physical systems and information propagation in a network. In this model, the source of the information is analogous to the role of a heat source, and information flows from a node with higher temperature (who was influenced) to a node with lower temperature (non-influenced). The authors [228] developed three diffusion models for selecting the best marketing candidates (users) who will maximize information spread in a network, leading to influence. The three diffusion models developed in [228] involve undirected social networks, directed social networks, and directed social networks with prior knowledge of their diffusion probabilities.

The models reviewed above employ stochastic analyses [229,230], which estimate the probability that a user will spread a piece of information. Stochastic modeling has been established as a useful strategy to analyze OSNs [231,232,233,234].

Some studies have combined a topic model and information diffusion, for example, [33]. In contrast to structural information spread models, topic-aware models for explaining information spread in OSNs analyze textural information and treat the latent topics identified in the texts as representing users’ interests [235,236]. In these models, topics represent the collection of a user’s interests that imply the user’s intentions to interact with other users [15,44]. For example, the authors in [235] proposed a mixed latent topic model to predict users’ re-posting behaviors. They assumed that users’ posting behavior is influenced by three factors: breaking news, posts from OSN friends, and users’ personal interests. Some topic-aware information spread models [15,46] use the Latent Dirichlet Allocation (LDA) topic model [237] to designate that users with the same topic distribution share the same behavioral pattern (role). Most topic-aware information diffusion models consider the topics that a user engages with, but neglect the user’s structural attributes, for example, neglecting the effects of role-topic pairs on the information diffusion process.

Overall, the non-structural information spread models in OSNs reviewed so far focus on modeling the spread by all network users (not only neighbors) but largely ignore the structure of the network and, thus, do not differentiate between local and global exposures to information that can lead to local and global influence, respectively. As described in Section 4.1, structural information spread models focus on modeling only local information spread as a result of local user-to-user exposure while ignoring exposure effects by non-adjacent neighbors in the network. Detecting both local and global information spread while considering network structure in a hybrid model, as described in the next subsection, is crucial to better understand human behavior online that determines users’ roles [68].

### 4.3. Hybrid Information Spread Models

**Figure 5:**
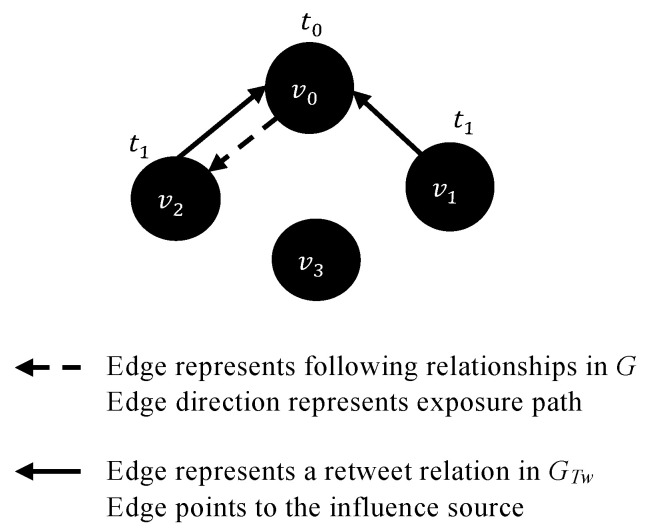
A directed social network G(V,E) (e.g., Twitter Following–Followee relationships) with a directed interaction network $G_{Tw}\left(V_{Tw},E_{Tw}\right)$ (e.g., retweets). The interaction network at time $t_{1}$ contains the set of nodes $V_{Tw}=\left\{v_{0},v_{1},v_{2}\right\}$, and the social network contains the set of nodes $V=\{v_{0},v_{1},v_{2},v_{3}$}. In *G*, node $v_{2}$ follows node $v_{0}$, indicated by a dashed edge (link). Thus, $v_{0}$ exposes $v_{2}$ to information. $v_{2}$ and $v_{1}$ retweeted $v_{0}$’s original tweet at time t1, indicated by two solid edges.

Bartal et al. [68] identified user exposure to local information spread in a dynamic OSN by considering network structure, as well as exposure to global information spread by non-neighbors. Thus, the authors extended and improved the existing network information spread models that can explain influence spread in OSNs. In another study, Bartal et al. [19] empirically detected local and global exposure to information that can lead to local/global influence in the form of retweeting a message on Twitter, considered a directed OSN G=(V,E) in which nodes are users and edges represent following relationships among users. Then, the authors tracked the temporal retweet sequence of an original tweet by users in *G* who were represented as nodes in a temporal interaction network $G_{Tw}=\left(V_{Tw},E_{Tw}\right)$ in which edges represent retweeting activities. Overlaid together, the social network *G* and the interaction network $G_{Tw}$ allow the detection of local and global information spread that resulted in influence events. *Local influence* was detected if a user retweeted an original tweet after one of the users whom s/he follows had retweeted or posted an original tweet. *Global influence* was detected if a user retweeted an original tweet before any of the users s/he follows had retweeted or posted it. Figure 5 illustrates an example of how local and global influence were detected in [19]. Given a social network *G* and an interaction network $G_{Tw}$, user $v_{0}$ posted an original tweet at time $t_{0}$, exposing user $v_{2}$ who follows $v_{0}$. Then, at time $t_{1}$, $v_{2}$ retweeted the original tweet that was posted by $v_{0}$, demonstrating local influence since $v_{2}$ follows $v_{0}$ in *G*. At time $t_{1}$, user $v_{1}$ also retweeted the original tweet that was posted by $v_{0}$, thus demonstrating global influence since $v_{1}$ does not follow $v_{0}$ in *G*. User $v_{1}$ might have been exposed to the tweet posted by $v_{0}$ via, for example, Twitter’s content recommender algorithm, or actively browsing for information.

Cha et al. [238] also studied the spread of information that is not limited to user-to-user spreading mechanisms. The authors analyzed the different roles (influential users, and ordinary users who became influentials) that users play in OSNs by analyzing (i) user popularity via Indegree network centrality; (ii) information spread of a specific topic measured using retweeting activities; and (iii) value of a user in an OSN via mentioning activities by other users.

Additional studies have modeled the spread of information beyond internal network sources of information. For example, Myers et al. [27] focused on exposure to viral information that spreads to multiple network users in a short period of time via information sources that are located outside of the network such as the mainstream media. More specifically, as mentioned above, they measured the level of exposure to information using hazard functions that facilitate exposure curves, such that each new exposure increases the probability of influence. Leskovec et al. [239] analyzed a collection of 90 million articles and tracked how information in the form of phrases or memes spreads through the 1.6 million mainstream media sites and online blogs assessed. Their study presents a quantitative analysis of how global news broadcasts spread between mainstream and social media. The authors found that there is a lag of 2.5 h between the peaks of attention to a phrase in the news media and in blogs. Regarding topics, the Topical Role Model (TRM) presents a hybrid approach [240] that analyzes how topical interests affect the information spread process and also considers user roles to explain information spreading patterns in OSNs. The TRM model assesses the role-aware topic-level diffusion analysis, which emphasizes the interplays between user role-topic pairs and their influence on information diffusion. Another non-structural information spread model in OSNs applied a logistic model [40] that predicts influence by mainly focusing on temporal and topological dynamics. As opposed to other non-structural models that ignore the structure of the network, this model takes into account one topological feature that is the distance from a user who holds an infecting role (the influence source) to a user who holds an infected role, for detecting information spread that resulted in influence.

Table 2 presents a summary of the main studies about information spread in networks that are covered in the current survey. The studies are grouped into three information spread research approaches: local, global, and external information spread. In this review, ‘external’ information spread sources are located outside of the network, whereas ‘global’ information spread sources are network nodes that expose non-neighboring nodes. Hybrid models span both local and global information spread models.

Whereas local influence occurs following exposure to information by network neighbors, global exposure results from exposure to information by non-neighbors and can lead to global influence. One way by which global influence can occur is by homophily between network users [18] and often requires extracting features of users [243], to apply machine learning algorithms for predicting information spread [22].

### 4.4. Homophily-Related Role-Aware Models

Homophily is defined as “the degree to which people who interact are similar in beliefs, education, social status”, and other characteristics [244] and has been found to increase the effectiveness of communication among individuals [245]. In other words, individuals prefer to associate with others whom they perceive to be similar to themselves in terms of both values and status characteristics that reflect users’ roles. Zhao et al. found that users with high homophily tend to have similar roles [122]. McCroskey et al. [245] viewed homophily as a four-dimensional construct, consisting of attitude, background, morality, and appearance. These dimensions of homophily include: (i) attitude homophily reflecting the extent to which a person perceives that another person shares his/her attitudes; (ii) background homophily indicating the extent to which a person perceives that another person shares his/her social background; (iii) value homophily denoting the extent to which a person perceives that another person shares his/her values and morals; and (iv) appearance homophily measuring the extent to which a person perceives that another person looks similar to him or her. Opinion leaders were found to have higher homophily than others on the dimensions of values, attitude, and background [245].

Similarity measures are typically used to quantify homophily among users and were able to explain the formation of relationships, and strength of interactions [246]. For example, Hanks et al. [247] found that perceived similarity to other customers in the services industries can influence both self-image congruence and self-brand congruence. Moreover, user similarity increases their commitment to the community and user perception of information quality [246,248]. Wang et al. [248] report how homophily plays an important role in determining credibility perceptions and influencing the persuasive process on both websites and online discussion groups.

Homophily affects people’s intentions of seeking opinions and consumers’ insights on social network sites. It may explain consumers’ reactions to online content generated by another consumer, opinion leader, or celebrity, such as a vlogger [249,250]. For example, consumers are influenced by recommendations from reviewers who are similar to them [249]. People feel similar to other people who reflect their own self-image [247]. Indeed, the more similar a person feels to another person, the more likely s/he will interact frequently with that person [251].

Previous studies show that homophily is an essential aspect in the study of OSN users [251,252]. YouTube viewers are more likely to recommend a vlogger and purchase the products featured in the vlog when they perceive that vlogger to be more similar to themselves [253]. Kim et al. [254] found that higher homophily between the consumer and the website lead to a positive attitude toward the website and toward the associated information posted on that website. Sakib et al. [255] found that homophily between a vlogger and his/her audience increased social interactions (in a context of weight loss).

The majority of recent influence spread studies in networks (e.g., [22,23,24,25,48,49,50,51,52]) focus on influence spread of viral information, but largely ignore influence spread of non-viral information, as discussed next.

## 5. Viral vs. Non-Viral Information Spread in Online Social Networks (OSNs)

In their study of viral information spread that produces influence via complex contagion, Romero et al. [16] evaluated users’ exposure to the 500 most frequent hashtags in a Twitter dataset consisting of over 3 billion messages. As mentioned above, the “Yes We Can” slogan [53] was employed in the 2008 U.S. presidential election in both mainstream and social media, initially by users who played the role of opinion leaders, and subsequently by standard users who served other roles. This slogan reached millions of voters and contributed to mobilizing millions of volunteers to actively support the ultimately successful presidential campaign [256]. The above are examples of viral information spread serving as the primary source of influence spread.

To date, most studies have focused on the spread of viral information in networks; however, no consensus exists among those studies regarding the number of influence events that define the virality of an information nugget, a unit of information such as a message or an image. For instance, Dow et al. [17] assessed a Facebook dataset consisting of approximately 1 million images, in order to study how influence diffuses via users’ activities to share images on that platform. The authors identified users who hold an influential role, using two linear regression models fitted with least squares that predict the importance of a node, defined by: (i) number of nodes who shared the information posted by the node in focus, and (ii) the size of the cascade generated by the node in focus. The linear regression results (maximal $R^{2}=0.49$) show that the audience size variable yielded the greatest explanatory power; other variables explained less than 1% of the variance. That study designated images that were shared a minimum of 100 times as being viral; this definition represents a fairly small number of influence events, relative to other studies. Providing an even broader definition of virality, Myers et al. [27] specified that posts shared at least 50 times are viral; this is one of the lowest thresholds of event counts among studies to detect influence. The authors presented a model in which information can reach a node via: (i) the links of the social network, modeled using a hazard function: $\lambda_{int}\left(t\right)dt\equiv P(i$ exposes j∈[t,t+dt)|i has not exposed *j* yet), where nodes *i* and *j* are neighbors, and *t* is the time passed since node *i* was infected. $\lambda_{int}$ represents the time it takes a node to observe an infected neighbor; or (ii) through the influence of external sources: $\lambda_{ext}\left(t\right)dt\equiv P(i$ receives exposure ∈[t,t+dt)); here *t* is the time passed since any contagion occurred. The authors found that approximately 71% of the information can be attributed to network diffusion, and the remaining 29% is due to exposure to information outside the network. Liben-Nowell and Kleinberg [49] quantified the depth reach of influence events involving the re-sharing of information by modeling the waiting time of a user before infection. The waiting time was modeled according to the density function $f\left(x\right)=x^{-\alpha }$. The authors found that events with broad reach can influence millions of users. In that study, the depth reach of influence events was measured starting from the originating user to the farthest user connected by a path of network edges to the originating user.

Numerous studies have reported that the number of users influenced by information nuggets follows a long-tailed distribution [22,54], that is, the majority of information instances are shared minimally [17,20,22,55], and most information nuggets are never shared. This observation suggests that the influence resulting from viral versus non-viral information spread occurs via different mechanisms that are detectable by observing the spreading patterns characteristic of these phenomena [19]. Furthermore, detecting these spreading patterns of influence can inform predictions during early stages of information transmission about whether an information nugget will go viral. For example, it was found [19] that the spread type of a contagion can be predicted in its early stages using two logistic regression models, one for viral (F1=0.84), and the other for non-viral (F1=0.87) information with Δt, the time difference from the posting of the original tweet variable, presenting the greatest predictive power. Other studies [23,48] developed a strategy to detect whether an information instance will become viral by assessing the reach size (or cascade) of information using data including hashtags [50], behavioral dynamics characteristics [24], and YouTube views [257].

Network features have also informed predictions of influence cascades’ reach size [22,52]. Gleeson and Durrett [258] reported that network structure and temporal dynamics are able to explain the observed spreading patterns of non-viral cascades. Cui et al. [51] developed a logistic model that employs the importance-rank of each network node, provided a list of users who were previously influenced. While these strategies are effective for predicting the reach size of information diffusion, the majority are unable to predict virality during the early stages of information spread.

This challenge of predicting whether an information nugget will become viral during the early phases of its transmission, before it has had the opportunity to become viral, was successfully addressed in [19]. In that study, the authors innovated a Back-In-Time (BIT) strategy to assess the spreading patterns of viral tweets during the time period before those tweets became viral. They subsequently compared the spreading patterns of viral tweets before they became viral with the spreading patterns associated with non-viral tweets. In that study, an information nugget was defined as viral if it had been shared a minimum of 100 times, and as non-viral if it had been shared 10 to 99 times [19]. Information nuggets that had been shared fewer than 10 times were excluded from that analysis because the influence spread signature of such instances was too low to permit the effective assessment of influence spread.

In more detail, the BIT strategy initially assesses a viral tweet back in time at a point when it had been non-viral, that is, it had been retweeted 10 to 99 initial times. These tweets that were rolled back in time are termed BIT-tweets. To test whether there are significant differences in the early spreading patterns of BIT vs. non-viral tweets, the authors plotted the empirical cumulative distribution functions (ECDFs) of the time differences between each two consecutive retweets (inter-retweet times) of BIT and non-viral tweets. A Kolmogorov–Smirnov test [259] revealed significant differences ($p_{value}\leq 2.2\times 10^{-16}$) in the distance (D-statistic) between the ECDFs of BIT vs. non-viral tweets. Thus, BIT-tweets and non-viral tweets were characterized by different temporal spreading patterns that were identified by comparison of inter-retweet times.

These authors [20] analyzed the spread of non-viral information on Twitter that can cause the spread of either local or global influence. They considered the topic of a tweet via analysis of users’ engagement in retweeting non-viral content, and they reported that influence spread both locally and globally. The percentages of events associated with global influence were discovered to vary by topic, ranging from 8% for topics related to Crime, to 28% for topics associated with Technology. Bartal et al. [20] reported that global influence is more likely to be a result of users’ exposure to information promoted by external sources that can include mass media, information searches via the Twitter search box, or content recommender algorithms.

Recommender systems aim to predict items or information that a user will enjoy consuming by providing users with a personalized experience [260]. However, these systems often narrow user exposure to several types of information termed ‘filter bubbles’ [38] and might lead users to ideological polarization due to repeated exposure to similar content [261]. While many recommender systems ignore unpopular or newly introduced content, some systems encourage the recommendation of unpopular content [262]. These unpopular content items affiliate with the long tail of information distribution where most information is unpopular, and the minority of information is highly popular [22,54].

**Figure 6:**
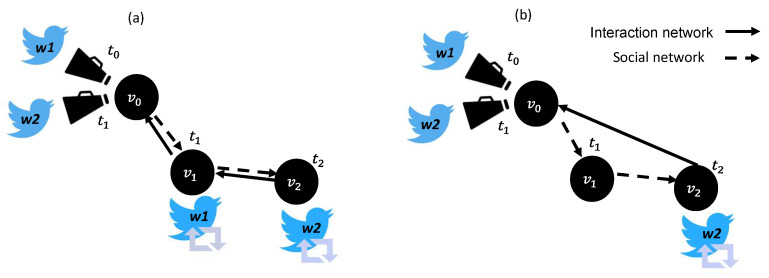
An illustration of local and global topic influence. Local and global topic influence of a set of tweets on the same topic $W=\{w_{1},w_{2}\}$ that were posted by user $v_{0}$ at times $t_{0}$ and $t_{1}$ respectively. (**a**) Local topic influence: user $v_{1}$ who follows user $v_{0}$ retweets $w_{1}$ at time $t_{1}$. Another example of local topic influence occurs after user $v_{2}$ was exposed to $w_{1}$ at $t_{1}$ by user $v_{1}$ whom s/he follows. Then, user $v_{2}$ retweets tweet $w_{2}$ on the same topic as $w_{1}$. (**b**) Global topic influence: user $v_{2}$ retweets $w_{2}$ before any of the users who are followed by $v_{2}$ retweeted/posted tweet from *W*.

Local and global influence spread following exposure to viral and non-viral topics on Twitter was examined in greater depth in [19]. In their analysis, a topic’s influence spread was detected when an individual user tweeted several messages about the same subject, and each tweet was retweeted by various other network members. In this study [19], a topic’s influence was defined somewhat differently than the influence of an individual information nugget: given a social network *G*, an interaction network $G_{Tw}$, and a set of tweets *W*, posted on the same topic by user $v_{j}$: (i) *Local topic contagion* of user $v_{i}$ occurs if $e_{ij}\in E_{Tw}$ and $e_{ji}\in E$. In other words, $v_{i}$ retweeted w∈W after one of the users s/he follows had retweeted or written any w∈W; and (ii) *Global topic contagion* of user $v_{i}$ occurs if $e_{ji}\notin E$ and $e_{ij}\in E_{TW}$. That is, $v_{i}$ shared w∈W prior to when any of the users s/he follows posted or shared any w∈W. Figure 6 illustrates local and global topic influence processes by which a user can be influenced by different tweets on the same topic.

In their study, the virality of a topic was predicted by summing the number of retweets of each tweet addressing the same topic that were posted by an individual user. While this study reported evidence of global influence, the authors reported that local influence was the most frequent infection mechanism for the spread of both a single information nugget and a topic [19]. In addition, viral information and viral topics were characterized by similar influence spreading patterns. Similarly, non-viral information and non-viral topics had similar spreading patterns of influence [19]. In contrast, viral and non-viral information and topics exhibited substantially different spreading patterns from each other, and local and global influence spread patterns were also seen to differ significantly [19].

The analysis of role-aware information spread in OSNs typically involves the development of software, and many platforms have been developed to explore user roles in information spread in OSNs, as discussed next.

## 6. Software Platforms for Role-Aware Analysis of Online Social Networks (OSNs)

Reviewing the literature, we differentiate between two types of software platforms for the role-aware analysis of OSNs, focused on an egocentric perspective of a single user and a global view of the network as a whole. The following section presents recent platforms exemplifying both analytical approaches (Table 3).

### 6.1. Platforms for Egocentric Role-Aware Social Network Analyses

In contrast to whole-network analyses, egocentric analysis involves personal communities related to single individuals, permitting detailed insights into “network neighborhoods” [263]. The focus of egocentric analysis is on a single central node (the ego) and its associations with its neighbors (alters). As an early example of role-aware information spread in OSNs, Xiong and Donath [264] created PeopleGarden, a platform that provides data portraits of users. Each user is represented by a flower-like icon, the properties of which are defined by the quantity and characteristics (initial posts vs. replies) of their messages. The roles of community members, and their relationships, are easily visible using these flower representations. Communities of users are represented as "gardens" of these icons, and can reveal high-level characteristics of OSN groups. PeopleGarden permits analysts to identify experts in the community, determine whether the group welcomes newcomers, understand the involvement of individual group members, and assess the overall activity patterns of the group.

A more recent example includes the D-map+ [265] algorithm that provides an integrated egocentric and event-centric model to interactively explore user behaviors and information spreading patterns. The platform’s egocentric analysis involves collecting data for the users who re-posted a message shared by the central user (ego) of interest. In contrast, its event-centric analysis focuses on one particular event, and examines all of the users associated with sharing and re-posting messages related to that event. D-map+ visualizes OSN users as hex nodes, with their behaviors and roles encoded by color and size. This platform permits the visual identification of important users and events.

**Dynamic Analyses.** Some of these platforms permit dynamic analyses of ego-networks. The Episogram software of Cao et al. [266] represents the agents and objects involved in interaction processes as a dynamic tripartite network, permitting the visualization of behavior patterns. This platform permits the identification of initiators and responders participating in interaction processes. Adopting a user-centric strategy, VASABI [267] provides dynamic hierarchical user profiles using data collected during OSN usage sessions, in addition to tasks extracted via topic modeling. This platform permits analysts to stratify user behavior and view high-level summaries of trends, and analysis can be performed at the levels of individual users and groups.

To permit the visualization of evolutionary influence graphs, Eiffel [268] provides visual summaries of influence graphs based on individual nodes, node relations, and temporal analyses. The relationships between an influencer of interest and the other categories of users whom they influence are presented in a novel flow map visualization that supports both flip-book and movie-based evolutionary visualizations to explore graph dynamics.

**Anomaly Detection.** Some egocentric visualization platforms are specifically designed to recognize anomalous user behaviors, which can be used to identify bots and other malicious entities in OSNs. The TargetVue platform of Cao et al. [269] is intended to detect anomalous users using unsupervised learning. The platform provides three novel glyph visualizations that present the ego node’s features, communication activities, and social interactions. These glyphs are arranged on a triangle grid that visualizes similarities across users and permits comparing the behaviors of users. The authors demonstrated the power of this platform to detect bots on the Twitter platform.

The egoStellar platform [270] provides the ability to analyze the communication behaviors of mobile users via an ego-network perspective; a specific objective of this platform is to identify anomalous behaviors and associated fraudulent and solicitor users. Three views are provided in its graph model, including a high-level statistical view to display the distribution of mobile users; a group view that permits classifying users and extracting features for anomalous behavior detection; and an egocentric view that details the interactions of each ego and its alters. More recently, the egoDetect platform [271] provides a novel visualization system for anomaly detection. This platform uses unsupervised machine learning that offers efficient anomaly detection without the need for training. The novel glyph visualization provided by this network permits the exploration of each ego’s topology and its relationships with its alters. Specifically, this platform permits the identification of different categories of alters, including local alters and alien alters, with the latter category indicating agents exhibiting anomalous behaviors.

### 6.2. Additional Platforms for Role-Aware Social Network Analyses

Additional platforms provide role-aware analysis of OSNs from other, more holistic perspectives than egocentricity (Table 3). Developed for use by service providers who wish to understand the characteristics of their users, iVIS [272] provides interactive visualizations of behavioral patterns and clustered views of behaviors. It permits the identification of light vs. heavy users, as well as more descriptive user categories including testers (those who used the service only for testing purposes) and frustrated users (those who examined a site’s data, but did not ultimately use it).

**Table 3:** Software platforms for role-aware analyses of online social networks. Rows are sorted primarily by year of publication, and secondarily by platform name.

Platform	Description	Publication Year	Reference
egoDetect	Detect and explore anomalies (alien alters) via unsupervised learning; novel glyph for ego topology	2020	[271]
Eiffel	View nodal, relational and temporal dimensions of evolutionary influence graphs to see influencer effects on other users	2020	[268]
iVIS	Identify light/heavy users and user categories via clustering	2020	[272]
VASABI	Analyze dynamic hierarchies at individual and group levels to identify user roles	2019	[267]
D-map+	View egocentric and event-centric information diffusion patterns; identify behaviors and roles	2018	[265]
egoStellar	Visualize anomalous users and behaviors via egocentric perspective	2018	[270]
MessageLens	Analyze learner attitudes, interactions among students, and discussion topics	2018	[273]
VisForum	Explore user groups in forums; identify high-impact forum members	2018	[274]
iForum	Analyze users, posts, and threads on three different scales; identify new, active and inactive users	2016	[275]
Episogram	Display dynamic egocentric social interactions to identify initiators and responders	2015	[266]
TargetVue	Identify anomalous users and behaviors via glyph visualizations	2015	[269]

VisForum [274] enables analysts to assess users and their roles in online forums, by presenting three novel glyph visualizations, at the group, user, and set levels, with different granularities. Their novel sorting algorithm reduces noise in the data, and their forum-index concept is used to identify high-impact forum members. For analyses focused on education, numerous platforms have been developed to understand the behavior and roles of course discussion forums [276]. Analytical objectives can include predicting students’ performance in courses, understanding sentiments, and predicting patterns of social behavior [276]. Examples of software for the prediction of behavior include the iForum platform [275], which permits the analysis of users, posts, and threads on three different scales, ranging from the complete forum down to the level of an individual thread or user. This platform allows the identification of various categories of users, including new users, active users, and inactive users. Providing a different perspective, the MessageLens visual analytics platform [273] allows instructors to understand their course forums based on discussion topic, interactions among students, and learner attitudes, permitting students to be classified by their behaviors.

The software platforms presented in this section, and listed in Table 3, permit the efficient role-aware analysis of information spread in OSNs. Establishing a standard to make this software more freely available would help to advance research on the behaviors and roles associated with complex human networks.

## 7. Conclusions and Future Directions

This review has surveyed the state of research on the role-aware analysis of information spread in online social networks (OSNs). Previous reviews of information spread in OSNs have mainly addressed local user-to-user propagation of viral information; however, recent studies have reported the importance of global spread mechanisms and their associated influence, and the impact of non-viral information spread. This review has addressed those gaps by: (i) providing a comprehensive survey of the latest studies on role-aware information spread in OSNs, describing the different temporal spreading patterns of viral and non-viral information and how user roles affect the spread of information; (ii) surveying modeling approaches for information spread in OSNs that consider structural and non-structural features as well as recent hybrid models that integrate both strategies, and presenting an associated taxonomy; (iii) providing an overview of software platforms used for the analysis and visualization of role-aware information spread in OSNs; and (iv) describing how role-aware information spread models enable useful applications in OSNs such as detecting influential users.

The current state of research on the role-aware analysis of information spread in OSNs suggests many promising future directions that call for future research, as described next.

**The Internet of People (IoP).** The Internet of Things (IoT) paradigm [277] provides a framework connecting the devices, sensors, actuators, protocols, and cloud services that have become important elements in managing the daily modern life. However, this paradigm is infrastructure-centric while regarding the human users as peripheral, thus neglecting an essential component of the system. To address this oversight, Conti et al. [278] have proposed an Internet of People (IoP) paradigm in which humans and their devices are active elements; the IoP paradigm is intended to enhance and extend, rather than replace, the existing IoT infrastructure [279]. In this framework, Dunbar’s ego-network model [280,281] can be used to map each individual’s social network to a concentric circle specifying different levels of membership, including the inner support clique, an intermediate sympathy group, a larger affinity group that is composed of an extended set of more distant friends, and an active network composed of 150 alters with which the individual maintains annual contact [278]. This model, originally developed to analyze real-world relationships, has subsequently been reported to usefully represent online social relationships, as well [282]. By accounting for these relationship structures, information diffusion in OSNs has been effectively modeled [283].

A central feature of the IoP is that human behavior models are embedded in its algorithms, requiring a broad integration among disciplines including psychology, sociology, anthropology, economics, and the science of complex networks [279]. Efforts to accurately represent the complexity of human behavior in OSNs via the integration of these disciplines are in their early stages. Continuing research on the behaviors and roles of OSN users will certainly inform the future developments of the Internet of People.

**Software Availability.** Unfortunately, the majority of the software platforms listed in Table 3 and described in Section 6 are not freely available; this prevents the widespread use of these platforms to inform research. Looking forward, it will be ideal if funding agencies require the open sharing of publicly funded software platforms, to ensure that these resources will be widely available to advance our understanding of the behavior and roles of individuals in OSNs.

**Visualization.** Novel immersive, interactive virtual reality (VR) and augmented reality (AR) visualization strategies have recently been revolutionizing many disciplines and fields of research [284,285]. While the full potential of the nascent VR and AR technologies has yet to be realized [286], the use of these technologies for network analysis offers a number of benefits. Three-dimensional (3D) VR representations of networks are reported to provide numerous benefits when compared with classic 2D networks [287], including facilitating accurate distance assessments between nodes [288], path finding among large numbers of nodes [289,290], community detection in complex graphs [291,292], and facilitating the accuracy of users’ mental models [293]. VR and AR platforms also lend themselves to collaborative work [294,295,296,297], including the creation of a 3D layout of a large Twitter network that provides collaborative interactions [298].

In recent years, these visualization strategies have started to be applied to the analysis of OSNs. The VRige platform [299] permits social network interactions to be explored in an immersive virtual environment. The VR-based framework of Sorger et al. [300] provides two navigation modes, overview exploration and immersive detail analysis, to analyze large dynamic networks. Looking ahead, the new field of cross-virtuality [301] has the goal of seamlessly integrating and transitioning among conventional 2D visualizations, augmented reality, and virtual reality; such integrative implementations have not yet been explored for the analysis of OSNs, and these represent an interesting future direction. Ens et al. [294] suggest that the use of immersive analyses might change the analytical process itself, promising novel strategies for the analysis of OSNs. Among these possibilities, integrating machine intelligence with human intelligence in immersive environments [294] promises to reveal new pathways for exploration of and discovery in OSNs. Finally, and importantly, to date, efforts to use VR and AR for network analysis have only recently started to include a role-aware focus [287], and this remains a promising and potentially very helpful direction for future work.

**Understanding the Complexity of Human Behavior Online.** As discussed in Section 2, OSN users’ roles are dynamic and varied. Humans are complex agents whose personality traits [302,303,304,305], habits [306], preferences [307], and interests [304,308] can evolve over time, and may vary based on a variety of factors. To date, role-aware analyses of OSNs have captured only a fraction of the complexity of dynamic human personality and behavior, and future research will benefit from considering additional facets of these phenomena to achieve more accurate predictions.

Many studies have developed strategies to identify anomalous behaviors for the purpose of eliminating malicious users and improving the social network experience for community members [309,310,311,312,313,314]. Rather than online behavior that is labeled as antisocial being limited to a small, vocal group of provocateurs, recent research has reported that standard OSN users can be provoked into becoming trolls or engaging in other antisocial behavior, influenced by the user’s mood and the context of the discussion [315]. Similarly, communications labeled as hate speech can be triggered in users by their OSN community members’ behaviors [316,317] as well as being more likely when specific topics are discussed [318,319]. Such research suggests that at least a subset of OSN users perceived as malevolent might become more positive contributors to OSNs by the thoughtful shaping of online environments.

Differentiating among various forms of behaviors labeled as antisocial in OSNs [315] will permit further granularity and informed control when managing these networks. In this vein, analysis to understand users’ intentions can inform the effective management of OSNs. For example, a user who posts messages that are assessed to be trolling content could have malicious intent, or might simply be expressing a sincere opinion that differs from those of the other forum members [315,320]. Additionally, users banned from OSN communities have been reported to form two distinct groups: those whose posts were regularly deleted by moderators prior to being banned, and those whose posts were only recently deleted before they were banned [321]. Extending research to differentiate habitual trolls from users who may have engaged in a specific heated debate [321] would be helpful to moderate online communities effectively, to avoid deplatforming users who do not habitually cause problems. Because the demographics, experiences, beliefs, and customs of OSN users can vary widely across OSN platforms, the definitions of social acceptability and toxic content differ by platform [322,323,324]. Accordingly, toxic content classifiers that use a single set of rules regardless of the OSN community will not capture the nuance of individual communities, and work is ongoing to tailor such standards to different groups of users [322]. Accurately characterizing users’ behaviors [325,326] is necessary to define roles and manage online communities effectively. Because ignoring the perspectives and needs of stakeholders in online communities can result in significant problems [323], it is important for role-aware OSN analyses to consider the perspectives of individual users, to ensure that each community member has an opportunity to participate and to feel that s/he is being heard. Ongoing work to understand and define social contexts [327], social acceptability [327,328], and the roles of OSN users will guide efforts to effectively manage OSN communities.

As discussed in the Internet of People subsection above, the coordination of network science with diverse fields including psychology, sociology, anthropology, and philosophy will allow us to achieve a deeper understanding of individuals’ behavior in interacting with information and other users in OSNs. Insightful integration of research across these diverse disciplines will facilitate network science, with a role-aware focus, to develop and moderate OSNs that will maximally benefit each individual community member.

## Figures and Tables

**Table 1:** Centrality Measure Algorithms for Opinion Leader Identification.

Centrality Metric	Algorithm Description	Citation
Betweenness$(v_{i})=$ $\sum_{v_{k}\neq v_{j}\neq v_{i}}\frac{\sigma_{v_{k},v_{j}}\left(v_{i}\right)}{\sigma_{v_{k},v_{j}}}$	Given a graph G=(V,E), $\sigma_{v_{k},v_{j}}$ is the number of shortest paths from $v_{k}\in V$ to $v_{j}\in V$, and $\sigma_{v_{k},v_{j}}\left(v\right)$ is the number of shortest paths from $v_{k}$ to $v_{j}$ that pass through a node $v_{i}\in V$.	[174]
Closeness$(v_{i})=$ $\sum_{v_{i}\neq v_{j};v_{i},v_{j}\in V}\frac{1}{\sigma_{v_{i},v_{j}}}$	Measure how close a node $v_{i}\in V$ is to all other nodes. Ranges from 0 (far) to 1/(|V|−1) (very central). σ is defined as in Betweenness.	[85]
Degree$(v_{i})=$ $\sum_{j}a_{ij}$, $a_{ij}\in A$ defined in Equation (1)	Number of edges connected to a node v∈V, or the number of neighbors of a node. Indegree - number of edges connecting into a node in a directed graph. Outdegree - number of edges going out of a node in a directed graph.	[175]
HITS $h\left(v_{i}\right)=\sum_{v_{j}\in V_{from}}a(v_{j})$ $a\left(v_{i}\right)=\sum_{v_{j}\in V_{to}}h(v_{j})$	In the HITS algorithm (hubs and authorities), each node $v_{i}$ has both a hub score $h(v_{i})$ and an authority score $a(v_{i})$. We initialize $a(v_{i})=$ $h(v_{i})=1$. $V_{from}$ - all nodes that $v_{i}$ links to. $V_{to}$ - all nodes linking to $v_{i}$.	[176]
H-index$(v_{i})=$ $H\left(k_{j}\right),j\in \Gamma_{i}$	Computes the interrelationships between publication quantities and numbers of citations, and defines a researcher’s academic influence in a particular domain. *H* is an operator on a set of real-valued variables $\left[y_{1},\ldots ,y_{n}\right]$ and returns the maximum integer *h* such that there are at least *h* members with a value (degree *k*) no less than h where $\Gamma_{i}$ is the set of neighbors of node $v{}_{i}$.	[177]
Extended H-index$(v_{i})=$ $\sum_{v_{j}\in \Gamma_{i}}CMC(v_{i})$	Utilize structural information from a node’s neighbors to compensate for the H-index algorithm that ignores network structure. The extended H-index considers the degrees of the neighbors of nodes $\left(\Gamma_{i}\right)$ using: (i) $S(v_{i})$ is the cumulative degree of the neighbors of node $v_{i}$; (ii) $S_{k}\left(v_{i}\right)$ is the value of the $k^{th}$ index of vector $S(v_{i})$; and (iii) $CMC\left(v_{i}\right)=\sum_{k=1}^{h}p^{1+k*\frac{p}{r}}\times s_{k}\left(v_{i}\right)$, where *h* is defined in H-index; and p,r∈[0,1] *r*.	[178]
Weighted H-index	A weighted H-index is calculated by constructing an operator *H* on weighted edges. The accumulation of weighted H-index in the node’s neighborhood defines the spreading, then utilizes the SIR model to investigate a spreading process, and define the most influential spreaders.	[179]
HybridRank$(v_{i})=$ $\sum_{v_{j}\in \Gamma_{i}}C(v_{j})\times EC\left(v_{i}\right)$ were $\Gamma_{i}$ is the set of $v_{i}$’s neighbors	Computes node’s importance using two centralities: (i) Eigenvector centrality (EC); and (ii) the Coreness (*C*) (see k-core in Table 1) sum of its neighbors. EC is a proxy of user influence in terms of connections with high-scored (central) nodes.	[180]
Improved HybridRank$(v_{i})=$ $\sum_{v_{j}\in \Gamma_{i}}C(v_{j})$ × H-index$\left(v_{i}\right)$	Combines two centralities: the Extended Neighborhood Coreness centrality and the H-index centrality. Then, it uses SIR.	[181]
k-core H⊆G,δ(G)≤k	The k-core of graph (*G*) is a maximal subgraph *H* in which each node has at least degree *k* (In or Out degree). The coreness of a node is *k* if it belongs to the k-core but not to the (k+1)-core.	[112]
Weighted k-core	Applies the same pruning routine as k-core, but measures both the degree of a node and the weights of its links.	[182]
LeaderRank $s_{i}\left(t+1\right)=$ $\sum_{j=1}^{N+1}\frac{a_{ij}}{k_{j}^{out}}s_{j}\left(t\right)$	The ranking process assigns 1-unit prestige to all (*N*) nodes in a directed network except the ground node (a node connected with every node). The unit prestige of the nodes is evenly distributed to neighboring nodes via links until a steady state is reached. Using random walks, the score of node *i* at time step *t* is $s_{i}\left(t\right)$; $a_{ij}$ is an element of an adjacency matrix.	[183]
Weighted LeaderRank $s_{i}\left(t+1\right)=$ $\sum_{j=1}^{N+1}\frac{w_{ij}}{\sum_{k=1}^{N+1}w_{jk}}s_{j}\left(t\right)$	Node ranking is calculated using two models: The first model measures the users’ relative influence based on quality of tweet, ratio of retweets, and topic similarity among users; and the second model calculates the user network global influence. This is an expansion of LeaderRank where the score from node *i* to node *j* is proportional to the weight $w_{ij}$ as defined in [184].	[184]
PageRank$(v_{i})=$ $\sum_{i\to j}\beta \frac{v_{i}}{d_{i}}+$ $\left(1-\beta \right)\frac{1}{\left|V\right|}$	Measures the importance of a node in a graph G(V,E) with |V| nodes, by counting the number of edges to a node to determine its importance. Important nodes are likely to receive more links from others. β: dumping factor; $d_{i}$: out degree of $v_{i}$.	[185]
VoteRank $S_{i}=\sum {}_{j\in \Gamma_{i}}V_{i}$ were $\Gamma_{i}$ is the set of $v_{i}$’s neighbors	Each node $v_{i}$ is represented by $\left(S_{i},V_{i}\right)$ where $V_{i}$ is $v_{i}$’s voting ability and $S_{i}$ is the score of $v_{i}$, i.e., the sum $S_{j}$ where $v_{j}$ is $v_{i}$’s neighbor. Initially, $V_{i}$ is set to 1. At each time step, $v_{i}$ with the largest score is selected into the target set, and then (i) the voting ability $V_{i}$ is set to zero; (ii) for each of $v_{i}$’s neighbors, its voting ability decreases by a factor $\frac{1}{k}$, where *k* is the average degree, and if $V_{j}<0$, we reset it as $V_{j}=0$.	[186]

**Table 2:** A summary of studies about information spread in networks that lead to influence, grouped by influence type.

Information Spread in the Study	Structural Models	Non-Structural Models	External Information
Resharing of online content (e.g., a message or a photo)	[21,22,49,50,202,241] [23,24,25,26,51]	[11,16]	[27,238,239]
Hybrid models of re-sharing online content	[19,20,40,68,165,240]		
Information first spreads to opinion leaders, and then from node to node	[30,164,203]		
Information on who adopted an innovation or behavior (e.g., prescribing a drug, imitation, or emotional contagion)	[199,201,210]	[18]	
Information on who adopted an innovation or behavior depending on the user’s number of adopting OSN neighbors	[22,52,206,207,209,242]	[41]	
Purchasing a product	[5,7,8,9,10,204]	[54,228]	

## Data Availability

Not applicable.

## Abbreviations

The following abbreviations are used in this manuscript:

2D	2-dimensional
3D	3-dimensional
AR	Augmented reality
BIT	Back-In-Time
BHLFM	Bayesian Hierarchical Latent-Factor Model
DAGs	Dynamic directed acyclic graphs
DMMSB	Dynamic Mixed-Membership Stochastic Blockmodels
DySTGNN	Dynamic structural-temporal graph neural network
GAT	Graph attention network
GCN	Graph convolutional network
GNN	Graph neural networks
IC	Independent Cascade model
IoP	Internet of People
IoT	Internet of Things
LDA	Latent Dirichlet Allocation
LIM	Linear Influence Model
LTM	Linear Threshold Model
LSTM	Long Short-Term Memory
MMSB	Mixed-Membership Stochastic Blockmodels
NLP	Natural Language Processing
NMF	Non-negative Matrix Factorization
OSN	Online social network
RNN	Recurrent neural network
RAFM	Role Affiliation Frequency Model
SVD	Singular Value Decomposition
SEIR	Susceptible–Exposed–Infected–Recovered
SIR	Susceptible–Infected–Recovered
SIRS	Susceptible–Infected–Recovered-for Susceptible
SIS	Susceptible-Infected-Susceptible
TRM	Topical Role Model
VR	Virtual reality
